# The social dimension of mindreading: developmental evidence for the role of social categorization during utterance interpretation

**DOI:** 10.1098/rstb.2023.0493

**Published:** 2025-08-14

**Authors:** Camilo R. Ronderos, Rebecca Iversen, Ira Noveck, Ingrid Falkum

**Affiliations:** ^1^ Center for Languages and Literature, Lund University, Lund, Sweden; ^2^ Department of Philosophy, Classics, History of Art and Ideas, University of Oslo, Oslo, Norway; ^3^ Laboratoire de Linguistique Formelle, Paris, Île-de-France, France

**Keywords:** experimental pragmatics, developmental pragmatics, social categorization, mindreading, Theory of Mind

## Abstract

Work in developmental pragmatics has shown that even though infants display refined mindreading abilities, older children struggle to understand language phenomena that rely on mindreading. This apparent mismatch might be partially explained by considering children’s growing sensitivity to social categories such as their interlocutor’s age. Based on recent work in philosophy of mind, we investigated how social categorization relates to children’s developing mindreading abilities during language comprehension. We tested the hypothesis that social-category-based reasoning follows a similar developmental trajectory to that typically described for children’s mindreading skills. In a picture-selection task, Norwegian participants (ages 3–9 years, *N* = 119) made decisions regarding a speaker’s (child or adult) preferences by choosing between images showing stereotypically child-coded and adult-coded items. Young children preferentially selected the child-coded image regardless of the speaker’s age, while older children selected the stereotypically adult-coded image when they heard the adult speaker. Additionally, we found no evidence that participants’ performance in the picture-selection task was causally predicted by their scores on a standard false-belief task. We interpret these results as suggesting that the use of social categorization skills during utterance interpretation describes a similar developmental trajectory to that typically described for mindreading abilities, but is likely independent from false-belief reasoning. We argue that future studies in developmental pragmatics should consider social category differences between participants and speakers when drawing conclusions about children’s mindreading abilities and how these are reflected in their interpretation of verbal utterances.

This article is part of the theme issue ‘At the heart of human communication: new views on the complex relationship between pragmatics and Theory of Mind’.

## 1. Introduction

Imagine that a 30-year-old watch salesman named Matías is speaking to a 90-year-old costumer named María Elena. During their conversation, María Elena says that she lost her wristwatch. Matías understands this to mean that she wants to buy a new one, so he shows her one of the watches on display. Matías is thinking about María Elena’s intentions so he can make sense of her (communicative) behaviour, and by doing so he engages in what is known as mindreading [1,2] or Theory of Mind [3]: the ability to track people’s mental states (in particular, their intentions, beliefs and desires) in order to interpret (and predict) their behaviour.

The idea that comprehenders reason about a speaker’s mental states when interpreting their utterances is at the heart of modern Pragmatics. Specifically, for researchers working within the Gricean paradigm, there is broad agreement that communication is successful when an addressee understands what the speaker wants them to understand and that they understand it (at least partially) because they recognize that the speaker intends them to do so [4–7]. What the speaker means to communicate is thus interpreted as an overtly expressed intention that is fulfilled when recognized by the other conversational participants [8]. Under this view, communication is tied to a comprehender’s mindreading abilities: the addressee interprets utterances as a function of what a speaker’s intentions are, which presupposes a disposition to think about the speaker’s mental states and interpret their behaviour on this basis.^1^

The theoretically stipulated link between utterance interpretation and mindreading has found empirical support in the adult language comprehension literature. For example, studies have shown that comprehenders are typically highly sensitive to a speaker’s identity, perspective, beliefs and desires when deriving meaning in real time [10–16]. Other studies suggest that people who score higher in mindreading tasks are typically better at understanding pragmatic language phenomena such as scalar implicature [17,18], metaphor [19], irony [20,21] and humour [22]. This all suggests that mindreading abilities play an important (and perhaps automatic; see [23]) role in facilitating various forms of successful communication for adults.

However, the literature on how mindreading skills develop with age (and how they relate to children’s developing pragmatic skills) is less clear. The standard view states that, before the age of four, children fail to explicitly attribute false beliefs to others ([3,24]; see [25] for a meta-analysis). This suggests that the spontaneous use of mindreading skills to interpret language use might be a late-emerging skill. More recent work, however, has argued that already at infancy children show sensitivity to the intentions and beliefs of others [26–30, i.a.]. By making the task more age-appropriate relative to the standard false-belief tasks (i.e. by implementing a more interactive story, introducing fewer characters to the story and changing the test questions to an open format), it is even possible for 3-year-olds to identify the false beliefs of others [31]. Furthermore, infants and preschoolers display refined pragmatic skills indicative of their mindreading abilities. For example, infants can detect intentional communicative cues [32] and consider the speaker’s intentions when learning new words [33,34]. Despite this apparent early attunement to other people’s mental states, preschoolers still seem to struggle to understand pragmatic language phenomena that rely on mindreading abilities, such as irony [35–38]. This mismatch between refined pragmatic skills in infancy (together with early sensitivity to others’ mental states) and poor performance of preschoolers in both false-belief tests and in understanding pragmatic phenomena that rely on mindreading is currently a highly discussed topic in developmental pragmatics [39–41]. What underlying factors give rise to this mismatch?

Before addressing this question, let us return to our initial example. Imagine that when Matías decides to show María Elena a wristwatch, he can pick from two new models that came in that day: an elegant, golden one or a plastic, Minions-themed one. Which one will he choose? If Matías presents her the golden wristwatch instead of the plastic Minions-themed one, he might be assuming that this is the watch that she wants based on what he believes to be appropriate for a woman her age. In other words, he would be predicting María Elena’s behaviour based on her age. The sorting of people and behaviour into social categories (such as age, race, gender or class) is known as social categorization [42].

This example shows how social categorization can play a role during mindreading: Matías attributes a desire to María Elena because of her age-based social category membership. This contributes to how he interprets and anticipates her behaviour. Such a relationship between social categorization and mindreading has been posited by philosophers of mind [2,43,44,45,46]. Spaulding [47] argues that the literature on mindreading has revolved around how people converge on interpretations of other people’s behaviour in the absence of a social dimension (such as when Matías infers that María Elena wants him to show her a wristwatch after she says that she lost hers). While mindreading convergence certainly takes place, Spaulding notes that focusing too much on it obfuscates the fact that people belonging to different social categories can (and very often do) diverge in how they interpret someone’s behaviour and on how their own behaviour is interpreted by others. For example, different inferences might be drawn from the same utterance depending on the speaker’s age. If María Elena were to be a child, Matías might instead try to sell her the Minions-themed watch. In other words, social categorization can be leveraged to ascribe mental states to others and subsequently to interpret and predict their behaviour. This form of interpreting behaviour therefore adds a *social dimension* to the traditional suite of mindreading abilities (i.e. the attribution of beliefs, desires and intentions). How we engage in mindreading—including the mental states we attribute to other people and the judgements we are predisposed to draw about them—is sensitive to the social categories we perceive them to fall into.

Returning to the question of how mindreading develops in children, imagine now that Matías has a 4-year-old son who is watching the interaction between his dad and María Elena and who can also see both available watches (the golden, elegant one and the plastic, Minions-themed one). When María Elena says she lost her watch, will Matías’ son also be able to infer that she would want to buy the elegant golden watch? More generally, to what extent is the development of mindreading abilities linked to how social categorization skills develop with age? Some work that (indirectly) speaks to this question has shown how a child’s social experience positively influences the development of their mindreading abilities [48,49]. Nevertheless, the social dimension of mindreading has otherwise been largely ignored, particularly when it comes to children’s developing mindreading abilities and their pragmatic language comprehension.

Stepping back from mindreading, it is important to note that there is a large literature in social psychology that does focus on how social categorization develops with age (for reviews see [42,50]). For example, it has been shown that infants are sensitive to social categories such as race, gender, attractiveness and native-speaker status [51–56]. Regarding the comprehension of age as a marker of social categories, it has been shown that infants display a preference to look at faces of other infants as opposed to those of adults [57,58]. Further work has shown that 2- and 3-year-old children often show a preference for their out-group (adults) over their in-group (peers) in situations where they require new information (e.g. [59,60]). However, other research on the same age group also suggests that children start having a more general social preference for their in-group. For example, Shutts and colleagues [61] showed that by the age of 3, children show a preference to play with an object that a child of their age has played with as opposed to one that an adult played with. This suggests that preschoolers start being influenced by the preferences of people whom they believe are part of their own social group around this age. It also suggests that age differences can be used to build social categories at an early age (on par with gender differences [61]). Furthermore, 3-year-olds are able to ascribe different domains of expertise to adults and children [62], suggesting that already at this age, children associate different traits with age-based social categories. Even though preschoolers already display a sensitivity for social categorization, their ability to use this skill to interpret their surroundings continues to change with age [50]. For example, 3-, 4-, and 5-year-olds prioritize belonging to a social category over personality traits when determining other people’s interpersonal preferences, whereas adults show the opposite pattern [63,64]. More generally, children’s assumptions that social categories are a stable component of someone’s identity, as well as their expectation that social categories are homogeneous, continue to develop throughout childhood (see [50] and the references therein). These findings are typically not framed in terms of mindreading. Some studies focus on how perception of social category membership influences the preferences of the children being tested (e.g. [57–61]), while others are interested in contrasting mentalizing-based and social-category-based inferences (e.g. [63,64]). However, a connection between social categorization and inferences about mental states can be found. VanderBorght & Jaswal [62], for example, argue that if preschoolers can ascribe different domains of expertise to adults and children, it presupposes that they can think about the (varying) epistemic authority of other people. Relatedly, Kachel and collaborators [59] see their findings as suggesting that children are able to infer the epistemic competence of their interlocutors based on age-based social categories. In both cases, one could argue that being differentially sensitive to the knowledge states of in- and out-group members requires that children represent these mental states in some way. Thus, both mindreading and social categorization skills seem to be working in tandem in these interactions, but this relationship is yet to be explicitly examined as it unfolds throughout cognitive development.

We see it as likely that at least some of these findings—for example, that 3-year-olds can identify age as a social marker and that they start displaying a preference for their social in-group in some circumstances [61]—have a bearing on how children use their mindreading abilities. For example, interpreting the behaviour of members of their age-based out-group (i.e. adults) might be different from how they interpret the behaviour of members of their age-based in-group (i.e. other children). This could, in turn, have repercussions for how preschoolers interpret utterances produced by adults (vs. children). Similarly, given how social categorization skills continue to change with age, it is reasonable to think that this developmental trajectory will also impact the development of pragmatic language comprehension: How a 3-year-old reasons about the communicative behaviour of adults vs. children might differ from how an older child does owing to changes in their social categorization skills. The importance of these observations becomes clear if we consider the fact that experiments in developmental pragmatics typically ask children to interpret the utterances made by an adult or by a puppet (who is likely to be understood by a child to represent a person [65]). This means that more often than not, studies in developmental pragmatics ask children to interpret the verbal behaviour of social out-group members, without a systematic investigation into how children differ in how they interpret the language of adults and children based on social characteristics. Thus, the role of social-category-based mindreading abilities in the development of pragmatic language comprehension remains underexplored, even though it is likely to influence how children engage in mindreading. The fact that social-category-based mindreading has not been considered in developmental pragmatics might therefore be one of the underlying factors that has brought about the apparent mismatch between early-developed mindreading abilities but late failures to understand mindreading-based pragmatic language.

In sum, we have so far raised the following issues: (i) There is an apparent mismatch between early-appearing pragmatic abilities in infants on the one hand and a failure to pass false-belief tasks (and understand non-literal uses of language at a later age) on the other. (ii) There is a gap in the mindreading literature when it comes to considering the role played by social categorization skills in interpreting the behaviour of others. (iii) This gap might be particularly important to consider for the development of mindreading abilities given how age-based social categorization skills develop continuously throughout infancy and childhood and how experiments in developmental pragmatics typically only examine children’s understanding of adult minds.

We therefore think that it is important for developmental pragmatics to take seriously the idea that interpreting behaviour based on social categorization is an essential part of how we think about other people’s minds. By addressing points (2) and (3) above, we will be better positioned to address point (1) and, ultimately, gain a richer understanding of the development of children’s pragmatic language comprehension skills.

The current study takes a step in this direction with two goals in mind. First, we aim to test the hypothesis that social categorization plays a part in mindreading as it contributes to how people attribute mental states to others and subsequently steers how behaviour is interpreted and predicted (as posited by [43,45,46]). We addressed this by studying the developmental trajectory of social-category-based mindreading: if reasoning about other people’s behaviour based on social category membership is part of mindreading, its development should follow the same trajectory typically described for mindreading abilities. Second, we aim to investigate the degree to which social-category-based mindreading skills are contingent on a child’s ability to attribute (false) beliefs to others. We addressed this by investigating the degree to which social-category-based mindreading is causally predicted by children’s performance on a standard false-belief task.

We pursue these two goals by way of a straightforward task that involves how children between the ages of 3 and 9 understand statements of a speaker’s preferences when expressed through possessive personal pronouns. For example, when an adult addressee hears a speaker say *‘This is my watch’*, that addressee is likely to be affected by the speaker’s social category: if the speaker is a middle-aged man, the addressee might anticipate the watch to be an elegant, golden one rather than a yellow plastic, Minions-themed one (when presented with both options). Children, on the other hand, might struggle to use their social categorization skills to predict the adult’s preference for a specific type of watch. Importantly, the child’s behaviour is likely to change as they grow older. The developmental trajectory of this change can be informative regarding whether social categorization-based inferencing is an expression of mindreading.

## 2. Method

### (a) Materials, design and procedure

**Figure 1.**
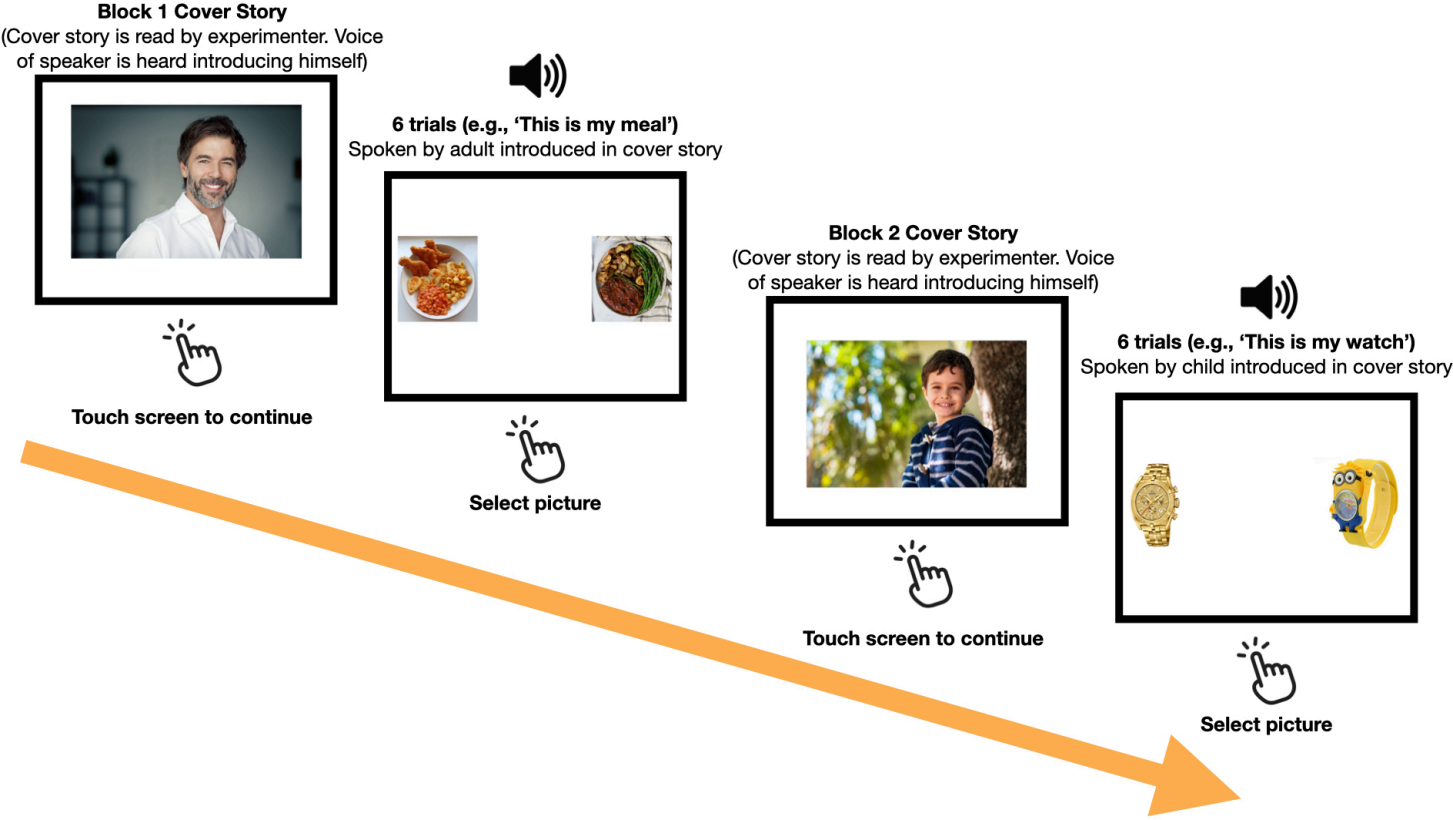
Sequence of events in an experimental session. Half of the participants saw the adult block first and the other half saw the child block first.

We created a picture-selection task, similar to those used in previous studies in developmental pragmatics (e.g. [66–69]), which are themselves part of the long tradition of referential communication experiments ([70,71]; [72], for a meta-analysis). In this task, we asked children to make a decision about a speaker’s preference. For example, participants heard a Norwegian speaker produce the Norwegian utterance *Dette er middagen min* (‘This is my meal’) and were required to choose between two possible (visually represented) options: one associated with an ‘adult’ social category (e.g. a fancy restaurant meal) and the other with a ‘child’ social category (e.g. chicken nuggets shaped like dinosaurs). The images we selected were matched closely for visual salience and size in order to avoid participants developing an associative picture-selection strategy (e.g. always selecting the more colourful picture of the two). The utterances all had an identical structure, varying only in the specific noun that the speaker described as being his (i.e. ‘This is my X’). There was a total of 12 critical items in the experiment. The experiment was programmed using PCIbex [73] and was run on a tablet computer. Participants were asked to touch the screen to select the picture that they felt best represented the speaker’s statement in each of the 12 trials. The sequence of events in the experiment is illustrated in figure 1.

The speakers of the 12 critical items were either a 4-year-old boy or a 50-year-old adult male. The items were introduced via two one-sentence cover stories prior to the trials, following a blocked, counterbalanced design that resulted in two experimental lists: one in which children first heard six adult trials followed by six child trials and another with the opposite sequence. The cover story showed a picture of the speaker together with his name and age. Participants heard the voice of the speaker introducing himself while they saw his picture (e.g. ‘Hi, my name is Tom, I am fifty years old’). Both speakers were white males and native speakers of Norwegian, and the entire experiment was conducted in Norwegian. The only salient feature that set the two speakers apart was their age.

As a consequence of having the two different speakers, our experiment had a within-subject, blocked factorial condition: SPEAKER AGE, with the levels ‘adult speaker’ and ‘child speaker’. A further (between-subject and continuous) condition was the PARTICIPANTS’ AGE, which was measured in days. A final independent variable came from participants’ scores in a standard false-belief task that was administered before the experiment (factor FALSE-BELIEF, levels: pass vs. fail).

### (b) Participants

We collected data from 119 participants (64 female, minimum age = 2.8 years, maximum age = 8.7 years, median age = 5.7 years). Children were recruited from kindergartens and elementary schools in the Oslo area, as well as from the Natural History Museum at the University of Oslo. All participants were monolingual Norwegian native speakers. We obtained written parental consent prior to testing. Each participant was tested individually in a quiet room at the museum, kindergarten or school. This study received ethics approval from Sikt - Norwegian Agency for Shared Services in Education and Research.

To determine the number of participants needed, we conducted a power analysis via simulations using the R package SIMR [74]. The power analysis was based on pilot data (*n* = 40) and concerned our predicted interaction between PARTICIPANT AGE and SPEAKER AGE (see §2e for details). For our power analysis via simulations, we used a conservative estimate of two-thirds the size of the effect found in the pilot study. With this new, smaller beta coefficient estimate, we simulated 1000 new datasets using the remaining parameters of the pilot model and re-ran the analysis on the new simulated data 1000 times. The results of the simulations suggested that with 100 participants, power would be over 80% to detect a true effect for the interaction between PARTICIPANT AGE and SPEAKER AGE. As an inclusion criterion, we required all our participants to accurately respond to the control question in the false-belief task (see §2d for details). Anticipating that we would lose some participants because of this, we tested 119 children. The final number of participants included in the analysis after exclusions was 100.

### (c) Picture norming task

Prior to data collection, we normed the experimental items on a sample of adult participants to make sure that the images we selected were typically associated with the correct social category (i.e. that the images we selected to represent things typically associated with adults and children were in fact perceived as such). We asked 83 adults (M = 25 years, SD = 8.3) to rate (on a 7-point Likert scale with 1 = completely disagree, 7 = completely agree) how much they disagreed or agreed that the pictures for 13 different test items were likely to belong to a 4-year-old or a 50-year-old. Each participant was randomly assigned to one of four lists that varied in terms of the stereotypical association of the item to an age group (two of the lists contained pictures of items typically associated with a child and the other two contained pictures of items typically associated with an adult), and in terms of the wording of the test question (in two of the lists the participant was asked how likely it is that the item belongs to a 4-year-old and in the remaining two how likely it is that the item belongs to a 50-year-old). These manipulations meant thatwe expected high ratings in two of the lists for each item (when the item belonged to the child/adult and the participant was asked how likely it was to belong to the child/adult, respectively) and low ratings in the remaining two (when the item belonged to the child/adult and the participant was asked how likely it was to belong to the adult/child, respectively).

For cases where the ownership of the item and the wording of the question were aligned, we determined that the item needed an overall median score of 6 or above to be included in the study. Moreover, when the ownership and wording of the question did not align, we settled on a threshold of a median score of 2 or below to be included in the study. In other words, we wanted to include only items that had both a strong association with a specific age-based social category and a strong disassociation with the other age-based social category. The results of the norming task led to one of the items being dropped, leaving the total at 12.

### (d) False-belief task

We used an adapted version of the ‘Birthday puppy’ story [75]. The adaptation included changing the linguistic control question to an ordinary first-order false-belief question. We also changed one sentence in the story from ‘I got you a really great toy’ to ‘I got you a basketball’, as this wording suited the illustrations better. The mindreading story was read out to the participants by an experimenter, and it was accompanied by five pictures that illustrated the story. In the story, Peter’s mother deliberately lies to him about his birthday present because she wants to surprise him. Later, unbeknownst to her, Peter discovers his present in the basement. When speaking to Peter’s grandmother, his mother is asked whether Peter knows what he is getting (second-order ignorance) and what he thinks he is getting (second-order belief) for his birthday. The story included two control questions (a reality control and a first-order ignorance) that the participants had to pass to be included in the analysis, and two test questions (first-order false-belief and second-order false-belief). Participants’ responses to both first-order and second-order false-belief questions were coded as 0 = fail, 1 = pass. Participants were also asked to justify their responses to the second-order false-belief question, but these responses were not included in the analysis. See the OSF repository for the full story script: https://osf.io/2y6cf/.

### (e) Predictions

Our main, pre-registered prediction concerned the developmental trajectory of social-category-based reasoning. If it is the case that using social categories to reason about a speaker’s preferences is linked to our mindreading abilities, we should observe the same developmental trajectory as is typically found for mindreading. The developmental trajectory of mindreading has been described as starting off with an ‘egocentric’ bias (or ‘true belief’ bias) [1,25,76–80]. That is, when children below the age of 4 fail the false-belief task, they do not make random mistakes. Instead, they systematically show that they ascribe their own belief to the agent in the story that they are asked to follow, suggesting that their starting point for reasoning about others’ beliefs is their own perspective (but see [81] for a different interpretation of the typical patterns found in the literature).

Thus, we predict that, in our experiment, we will observe a developmental trajectory such that young children will initially select the object that they themselves are likely to prefer (the object typically associated with children), regardless of the speaker’s age. Only as they grow older will they learn that adults' preferences typically prefer differ from children's. They should therefore continue to select the child-image only in the ‘child’ condition (i.e. when the speaker is a child). In the ‘adult’ condition (i.e. when the speaker is an adult), they should learn to identify the traits typically associated with the adult social category with age and eventually select the correct adult image. Concretely, this should result in an interaction between PARTICIPANT AGE and SPEAKER AGE.

Our second prediction involves children’s scores on the independent mindreading task and how these relate to children’s picture-selection pattern in our experiment. Since we hypothesize that social-category-based reasoning is part of mindreading, it could be that this is not only visible in its developmental trajectory (as our first prediction states) but also in how it relates to tasks that are typically used as an index of a child’s mindreading abilities, such as a standard false-belief task. For example, the development of social-category-based mindreading skills could be tied to how children begin to attribute beliefs explicitly to others. If this is the case, children who pass the first-order false-belief task will be more likely to correctly identify the adult image as the referent in the ‘adult’ condition (e.g. to reason that when the adult says ‘this is my meal’ he is talking about the meal typically associated with an adult). Meanwhile, children should be just as likely to correctly identify the child-image as the referent in the ‘child’ condition, regardless of whether they pass or fail the first-order false-belief task. The reasoning behind this prediction is that those who fail should deploy an ‘egocentric’ strategy and select the child-image by default. This pattern would translate to an interaction between the factors BELIEF-ATTRIBUTION and SPEAKER AGE. Alternatively, it could be that social-category-based mindreading abilities are not predicted by children’s performance in a false-belief task. If this is the case, we should find that participants’ scores on the ‘Birthday puppy’ mindreading task do not significantly predict the children’s picture-selection behaviour.

### (f) Analysis

Our analysis pipeline was pre-registered. The pre-registration form, analysis script and data are available on the project’s OSF repository: https://osf.io/2y6cf/.

All analyses were conducted using R [82] and R-Studio [83]. For data processing, visualization and analysis, we used the following packages: Ggplot2 [84], lme4 [85], Rmisc [86], MASS [87], Dplyr [88], doBy [89], Papaja [90], Here [91], emmeans [92] and sjPlot [93].

To test our hypotheses, we fitted two separate logistic regression models to the picture-selection data. In both models, the dependent variable was the picture selected, coded categorically (1 = adult picture, 0 = child picture). As per our pre-registration, the first model included the factor SPEAKER AGE (with the levels ‘child speaker’ and ‘adult speaker’, sum-contrast coded) and the continuous variable PARTICIPANT AGE (centred and scaled, measured in days), as well as their interaction. As a *post hoc* measure and for the sake of accuracy and completeness, we additionally included the control factors PARTICIPANT GENDER (two levels sum-contrast coded) and EXPERIMENTAL LIST (two levels, sum-contrast coded) as covariates. Including these control factors did not change the pattern of results for the critical interaction between SPEAKER AGE and PARTICIPANT AGE. The model also included a random intercept and random slopes for the factor SPEAKER AGE by participants, as well as a random intercept term and random slopes for the factors SPEAKER AGE, PARTICIPANT AGE (and their interaction), PARTICIPANT GENDER and EXPERIMENTAL LIST by items. This was the ‘maximal’ model given our experimental design [94]. The second model included the factor (first-order) BELIEF-ATTRIBUTION (two levels: ‘pass’ and ‘fail’, sum-contrast coded) and its interaction with SPEAKER AGE (included also as a random slope by items). As a deviation from our pre-registration, the factors SPEAKER AGE and PARTICIPANT AGE, together with their interaction, were included as controls. This was done to ensure that if an effect of BELIEF-ATTRIBUTION was found, it would not be caused by the mediating effect of the participant’s age, as well as by how PARTICIPANT AGE interacts with SPEAKER AGE (see [95] on why it is necessary to include such interaction effects as controls).

As an exploratory, *post hoc* test, we fitted a linear regression model that looked at the time it took children to select one of the images. The goal of this third model was to investigate whether the results of the first and second models could be related to potential differences in cognitive effort (as indexed by reaction times) when selecting an interpretation. This model included the factors PICTURE SELECTED (levels: ‘child picture’ and ‘adult picture’, sum-contrast coded), BLOCK (levels: ‘first’, and ‘second’, sum-contrast coded) and the three-way interaction between PICTURE SELECTED, SPEAKER AGE and PARTICIPANT AGE, as well as all other predictors present in the first model. The random-effects structure included random intercepts by items and participants, random slopes for PICTURE SELECTED, SPEAKER AGE and their interaction by participants, and random slopes for PARTICIPANT AGE, SPEAKER AGE, PICTURE SELECTED and all of the two-way interactions by items. We used the log-transformed reaction times as dependent variable, since the residuals of the model using raw-RTs were not normally distributed, and a box-cox test [96] suggested the log-transformation as the optimal one.

### (g) Results

**Figure 2.**
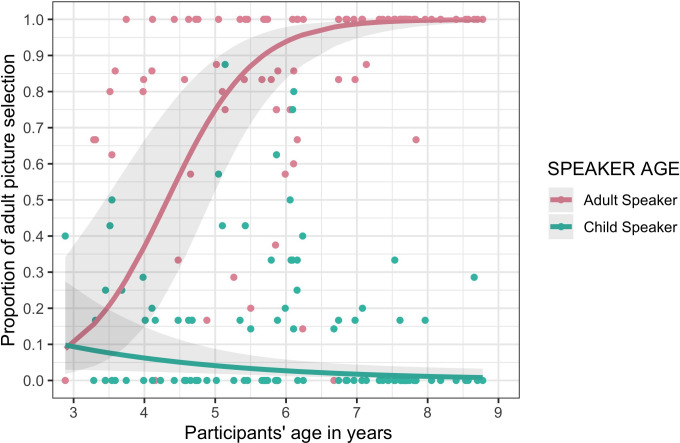
Proportion of participants who selected the adult image in the ‘adult’ speaker and the ‘child’ speaker conditions in the social-category-based mindreading task. Individual dots show participant averages. Plotted lines and grey ribbons show the predicted values of the logistic regression model and 95% CI, respectively.

**Figure 3.**
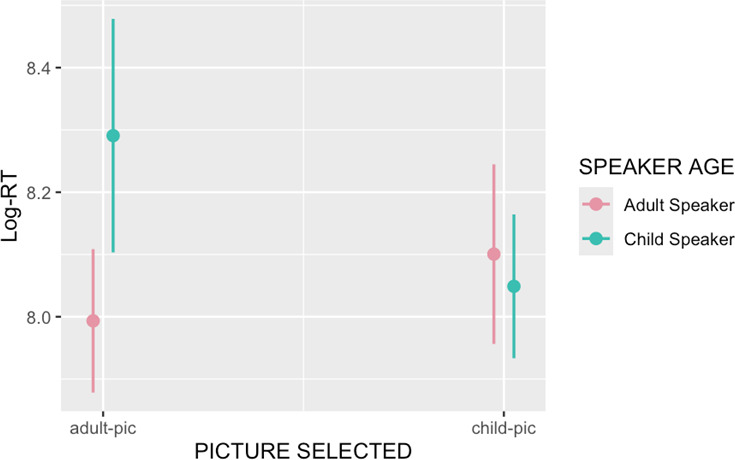
Model results for picture-selection time. Error bars show 95% CI.

The output of models 1, 2 and 3 can be seen in tables 1, 2 and 3, while the main findings are visually summarized in figure 2 and figure 3. As table 1 shows, there was a main effect of SPEAKER AGE (beta coefficient = 6.29, *z*-value = 9.46, *p*‐value < 0.001), showing that, across the board, participants selected the adult image in the ‘adult speaker’ condition and the child image in the ‘child speaker’ condition. There was also a main effect of PARTICIPANT AGE (beta coefficient = 0.89, *z*-value = 3.37, *p*‐value < 0.001), suggesting that older children tended to select the adult image more often than younger children did. This is critically accompanied by an interaction between SPEAKER AGE and PARTICIPANT AGE (beta coefficient = 3.11, *z*-value = 5.44, *p*‐value < 0.001), suggesting that the increase in adult image selection was due to participants’ performance in the ‘adult speaker’ condition: Children of all ages were similarly likely to select the child image in the ‘child speaker’ condition. Young children tended to select the child image in the ‘adult speaker’ condition, while older children preferred to select the adult image in the ‘adult speaker’ condition. This is in line with our first prediction. Model 1 also showed a main effect of EXPERIMENTAL LIST (beta coefficient = −1.05, *z*-value = −2.45, *p*‐value = 0.013), suggesting that in the first experimental list (in which the adult speaker appeared in the first block and the child speaker in the second block) participants selected the adult picture more often than in the second experimental list (in which the adult speaker appeared in the second block). There was no effect of the participant’s gender on picture selection. The output of the test of our secondary hypothesis is summarized in table 2. Importantly, thoughour initial, pre-registered analysis seemed to show an interaction between SPEAKER AGE and (first order) BELIEF-ATTRIBUTION, this interaction was actually no longer significant once the interaction between SPEAKER AGE and PARTICIPANT AGE was controlled for, as seen in table 2. The results of the exploratory third model (which investigated the children’s response times) showed a main effect of SPEAKER AGE, with pictures being chosen significantly faster when the speaker was an adult than when the speaker was a child (beta coefficient = −0.123, *t*-value = −2.41, *p*‐value = 0.0221). There was also an interaction between SPEAKER AGE and PICTURE SELECTED, which is visible in figure 3 (beta coefficient = −0.349, *t*-value = −2.88, *p*‐value = 0.0074). Finally, there were main effects of AGE (older children were faster to select a picture than younger children, beta coefficient = 0.235, *t*-value = −5.88, *p*‐value < 0.00001), and block (participants were faster to make a selection in the second block than in the first block, beta coefficient = −0.233, *t*-value= −8, *p*‐value < 0.00001). No other effects were significant.

**Table 1.** Summary of model output for picture selection.

term	$\hat{\beta }$	95% CI	z	p
SPEAKER AGE	6.31	[5.00, 7.62]	9.47	<0.001
PARTICIPANT AGE	0.90	[0.37, 1.42]	3.36	<0.001
participant gender	0.09	[−0.71, 0.90]	0.23	0.818
experimental list	1.05	[0.21, 1.90]	2.45	0.014
SPEAKER AGE * P. AGE interaction	3.12	[1.99, 4.25]	5.43	<0.001

Note. factors were sum-contrast coded, age was measured in days, centred and scaled.

**Table 2.** Summary of model output for effect of first-order false-belief score on picture selection.

term	$\hat{\beta }$	95% CI	z	p
SPEAKER AGE	6.56	[5.13, 7.98]	9.02	<0.001
BELIEF-ATTRIBUTION	−0.09	[−1.08, 0.89]	−0.19	0.853
PARTICIPANT AGE	0.92	[0.34, 1.50]	3.12	0.002
participant gender	0.10	[−0.75, 0.96]	0.24	0.811
experimental list	1.10	[0.17, 2.02]	2.33	0.020
SPEAKER AGE * BELIEF-ATTRIBUTION interaction	−0.72	[−2.96, 1.52]	−0.63	0.528
SPEAKER AGE * PARTICIPANT AGE interaction	3.09	[1.84, 4.34]	4.84	<0.001

Note. factors were sum-contrast coded.

**Table 3.** Summary of model output for picture-selection time.

term	$\hat{\beta }$	95% CI	t	df	p
PICTURE SELECTED	−0.07	[−0.17, 0.03]	−1.33	27.02	0.196
PARTICIPANT AGE	−0.23	[−0.31, −0.16]	−5.88	146.31	<0.001
SPEAKER AGE	−0.12	[−0.22, −0.02]	−2.41	30.33	0.022
participant gender	−0.07	[−0.21, 0.06]	−1.04	97.99	0.301
experimental list	0.12	[−0.02, 0.25]	1.66	98.62	0.099
BLOCK	0.23	[0.18, 0.29]	8.01	100.73	<0.001
PICTURE SELECTED * P. AGE interaction	−0.01	[−0.12, 0.10]	−0.15	20.23	0.879
SPEAKER AGE * PICTURE SELECTED interaction	0.35	[0.11, 0.59]	2.88	28.39	0.007
SPEAKER AGE * P. AGE interaction	−0.01	[−0.14, 0.12]	−0.17	15.16	0.869
three-way interaction	0.21	[0.00, 0.42]	1.96	86.55	0.054

Note. Reaction times were log-transformed. Factors were sum-contrast coded, age was measured in days, centred and scaled.

## 3. Discussion

In this study, we set out to do two things: probe the developmental trajectory of social-category-based reasoning and investigate the degree to which it relates to participants’ performance in a false-belief task. In terms of the developmental trajectory of social-category-based reasoning, we found a clear pattern indicative of children’s continuous improvement in using their social categorization skills to interpret the behaviour of adults: while 3- and 4-year-olds seemed to base their responses on their own perspective (and therefore selected the typical child image when they heard the adult speaker), older children consistently selected the typical adult image when they heard the adult speaking. When they heard the child speaker, however, participants of all ages consistently selected the correct child image, resulting in an interaction between the predictors SPEAKER AGE and PARTICIPANT AGE. This pattern confirmed our prediction that social-category-based reasoning about others’ behaviour displays the same developmental trajectory as has been described for mindreading abilities (such as ascribing false beliefs to others), with young children starting off with an ‘egocentric’ bias in which they initially ascribe their own perspective to other agents.

It could be argued that these results have a non-mentalistic interpretation. Young children in our experiment could have preferentially selected the child-coded picture simply because they are more familiar with the world of children than with the world of adults. As a consequence, they might have easily recognized the child-coded objects and wilfully selected them as targets regardless of the speaker’s social identity. When seeing the picture of a plate of dinosaur-shaped chicken nuggets, then, they might see it as an acceptable meal to have regardless of who the speaker is, whereas they might not have any associations with or experience of the adult-coded plate of food. Only as they get older do they gain experience of the adult world and become able to select the correct image, without needing to engage in any form of mindreading. Such an interpretation would make predictions regarding the time that it takes participants to select one of the pictures. It has been well established in the perceptual expertise literature, for example, that greater degrees of familiarity and expertise in a particular domain are inversely correlated with response times (e.g. [97–99]). Thus, we would expect children in our experiment to overall be faster at selecting the child-coded images than at selecting the adult-coded images, since they should be more familiar with the former than with the latter. The results of our experiment do not show this pattern. There is no significant difference in response times between selecting the adult-coded and the child-coded picture, and participants are overall faster at making a choice when they hear an adult speaking than when they hear a child speaking. There were also no interactions with the age of the participant, suggesting that participants behaved similarly across all ages. Taken together, this pattern of results makes it less likely that the deciding factor was children’s familiarity with their age peers. Additionally, there was an interaction between SPEAKER AGE and PICTURE SELECTED. Children who selected the adult-coded image when the speaker was a child took longer to do so than those who selected the correct, child-coded image, with the reverse pattern appearing for the adult-coded image. This suggests that there was added cognitive effort associated with a mistaken interpretation, providing further evidence for the idea that participants did think about the identity of the speaker when selecting the image, and did not simply select the child picture because they were more familiar with it.

Our results did not support our secondary hypothesis, according to which participants’ scores in a standard false-belief task should causally predict their performance in the picture-selection task. The findings showed that participants who passed the first-order false-belief test were better at using their social categorization skills to select the adult picture in the ‘adult speaker’ condition relative to those who failed the first-order false-belief test. However, this effect was not significant after controlling for the participant’s age and its interaction with the speaker’s age. This suggests that the age of the participants was the critical mediator:as they grow older, children become more proficient at ascribing false beliefs, as well as at deploying their social categorization skills. The lack of a significant effect (paired with our confirmed primary hypothesis and the reaction times results) could be explained when considering the stage in development tracked by a false-belief task. As pointed out in §1, there is evidence suggesting that children are capable of drawing some forms of mentalistic inferences before they pass the false-belief task (see [100] for a review of so-called ‘implicit’ and ‘explicit’ theory of mind tasks). It could therefore be that success in the false-belief task indexes an advanced stage in the developmental continuum of a child’s mindreading skills that is not needed for children to deploy their social categorization skills to interpret the behaviour of others. Alternatively, this finding can be interpreted as being in line with recent accounts that view mindreading as involving multiple different mentalistic representations that can vary greatly in terms of their processing cost and age at development [101]. In this sense, social-category-based mindreading could be developing in parallel with belief reasoning, without being contingent on it. Further experiments are necessary to investigate whether other types of mentalistic representations (and if so, which ones) need to be in place before children can use social categories to predict the behaviour of others.

Taken together, the pattern of findings provides developmental evidence for the relationship between social categorization and mindreading, consistent with what has been described in the philosophical literature (e.g. [47]). When thinking about other people’s mental states to interpret and predict their behaviour, we often rely on information available to us from the social context, such as features that we typically associate with different social categories (for example, age). This ability seems to develop ontogenetically in tandem with (but, perhaps, independently from) our ability to ascribe false beliefs to other people, and it describes a similar developmental trajectory to that typically associated with mindreading skills.

Our study builds on previous psycholinguistic work on the integration of a speaker’s identity during sentence processing. Van Berkum *et al*. [10], for example, found that hearing a sentence such as ‘Every evening I drink some wine before I go to sleep’ in a child’s voice (as opposed to an adult voice) elicited event-related brain responses within 300 ms after hearing the critical word *wine*. They interpreted their results as demonstrating the early role of pragmatics during online utterance interpretation, implicitly acknowledging the link between social categorization (e.g. if the speaker is a child or an adult) and mindreading (via the integration of a speaker’s intentions with the unfolding utterance). Our work complements this perspective by further showing that despite the fact that the attribution of mental states to others based on their social categories plays a rapid role during adult language processing, children only start displaying adult-like performance at around 5 years of age. Similarly, our work also complements psycholinguistic studies that show how the social characteristics of the speaker (such as native-speaker status) influence adult sentence processing (e.g. [102]) and, critically, how these social characteristics can lead people to derive pragmatic inferences at a lower rate when the speaker is a member of a social out-group (such as being a non-native speaker [103–105].

Besides empirically supporting the theoretical claims regarding the social dimension of mindreading, our study has potential consequences for open debates in developmental pragmatics. In particular, the results of our experiment suggest that studies in developmental pragmatics need to take into account social category differences between participants and experimenters. Concretely, future experiments should consider the impact of asking children to interpret language used by adults as opposed to that used by other children. Our current findings show that, under certain circumstances, children differ in their reasoning about the communicative behaviour of adults and the communicative behaviour of other children. Since this is part of children’s mindreading abilities (and it influences how they interpret other people’s behaviour), it is likely to influence how they interpret pragmatic phenomena that have been claimed to recruit mindreading abilities such as irony [15], metonymy [106], metaphor [107], humour [22], and even scalar implicatures [15,17].

We see at least one way in which social categorization could play a role during the development of pragmatic abilities. It could be the case that young children assume other children to behave like they would, seeing as, in our experiment, they appear to deploy an ‘egocentric’ strategy when making judgements based on social categories. As a result, they could be better at drawing inferences about the minds of members of their own social category (i.e. other children). This could lead to a better understanding of (some) pragmatic language phenomena when the speaker is a child vs. an adult. Such a result would be in line with studies that show that people are less likely to identify a sentence as being ironic when it is produced by a non-native speaker (i.e. an out-group member) than from a native speaker (i.e. an in-group member; e.g. [103]). However, exactly which pragmatic phenomena and how their understanding is (or is not) facilitated will have to be empirically tested. The social dimension of mindreading might thus be one of the missing puzzle pieces that can help us towards understanding why infants have been shown to be highly sensitive to others’ perspectives, and yet they ostensively fail to grasp phenomena such as irony until their school years [35,36,38], or seemingly fail to understand metaphors until 10 years of age [107,108].

## 4. Conclusion

The current study set out to investigate the hypothesis that the development of social categorization is connected to children’s development of mindreading abilities. Basing our hypotheses on insights from work in philosophy of mind [44–46], we found that social-category-based mindreading follows a developmental trajectory akin to that of other mindreading abilities: when listening to an adult man speak about items that belong to him, young preschoolers consistently ignored stereotypically age-specific preferences and instead selected items typically associated with children. Only older children showed sensitivity to the age-specific preferences and selected the appropriate adult-coded item. This pattern of results was not predicted by children’s scores on a false-belief task, suggesting that highly developed mindreading abilities (such as explicitly ascribing false beliefs to other people) might not be necessary in order for children to deploy their social categorization skills to draw inferences about the behaviour of others. Overall, these results might help bridge a gap in the literature on children’s development of communicative skills, raising the possibility that social categorization acts as a moderating factor in how children’s pragmatic skills develop with age. Future studies should examine the developmental trajectory of the social dimension in more depth and how it may impact children’s understanding of pragmatic phenomena, as well as whether other social factors play a similar role in the development of mindreading skills.

## Data Availability

Data and analysis script are avilable on the project's OSF repository: [109].

## Footnotes

^1^
See Sperber & Wilson [9] for a proposal that pragmatics is a specialized sub-module of mindreading abilities.
